# The relationship between heart rate variability and TNM stage, co-morbidity, systemic inflammation and survival in patients with primary operable colorectal cancer

**DOI:** 10.1038/s41598-023-35396-x

**Published:** 2023-05-19

**Authors:** Josh McGovern, Stephen Leadbitter, Gillian Miller, Adam Hounat, Irvine Kamande, Ross D. Dolan, Paul G. Horgan, David K. Chang, Nigel B. Jamieson, Donald C. McMillan

**Affiliations:** Academic Unit of Surgery, School of Medicine, University of Glasgow, Level 2, New Lister Building, Royal Infirmary, Glasgow, G31 2ER UK

**Keywords:** Surgical oncology, Prognostic markers, Cancer, Colorectal cancer

## Abstract

High vagal nerve activity, reliability measured by HRV, is considered protective in cancer, reducing oxidative stress, inflammation and opposing sympathetic nerve activity. The present monocentric study examines the relationship between HRV, TNM stage, co-morbidity, systemic inflammation and survival in patients who underwent potentially curative resections for colorectal cancer (CRC). Time-domain HRV measures, Standard Deviation of NN-intervals (SDNN) and Root Mean Square of Successive Differences (RMSSD), were examined as categorical (median) and continuous variables. Systemic inflammation was determined using systemic inflammatory grade (SIG) and co-morbidity using ASA. The primary end point was overall survival (OS) and was analysed using Cox regression. There were 439 patients included in the study and the median follow-up was 78 months. Forty-nine percent (n = 217) and 48% (n = 213) of patients were categorised as having low SDNN (< 24 ms) and RMSSD (< 29.8 ms), respectively. On univariate analysis, SDNN was not significantly associated with TNM stage (p = 0.830), ASA (p = 0.598) or SIG (p = 0.898). RMSSD was not significantly associated with TNM stage (p = 0.267), ASA (p = 0.294) or SIG (p = 0.951). Neither SDNN or RMSSD, categorical or continuous, were significantly associated with OS. In conclusion, neither SDNN or RMSSD were associated with TNM stage, ASA, SIG or survival in patients undergoing potentially curative surgery for CRC.

## Introduction

The autonomic nervous system (ANS) forms an intricate network of connections that maintain homeostasis via tightly regulated sympathetic and parasympathetic outputs^1^. The Vagus nerve, a major component of the parasympathetic nervous system, is thought to be protective in cancer^2,3^. Specifically, vagal nerve activity is thought to reduce oxidative stress and inhibit sympathetic nerve output^4^. Moreover, reduce inflammation via the cholinergic anti-inflammatory pathway^5^.

Heart rate variability (HRV) is defined as the variation in time interval between successive heartbeats^6^. Reported to be a reliable measure of vagal nerve activity^7^, recent systematic reviews have found an association between HRV and survival outcomes in patients with cancer^8,9^. Furthermore, HRV has been associated with prognostic factors associated with cancer outcomes including advanced age, co-morbidity, advanced disease stage and systemic inflammation^10–14^. As such, the basis of this relationship between HRV and survival outcomes in cancer remains unclear.

To date, the majority of studies examining HRV in colorectal cancer (CRC) have been carried out in patients with locally advanced or metastatic disease^15–18^. Indeed, there is a paucity of studies examining the relationships between HRV, tumour/host characteristics and survival in patients with potentially curative CRC^19^. Therefore, the aim of the present study was to examine the relationship between HRV, TNM stage, co-morbidity, systemic inflammation and survival in patients undergoing potentially curative resections for non-metastatic CRC.

## Methods

### Patients

Consecutive patients who underwent potentially curative resections for colorectal cancer, within NHS Greater Glasgow and Clyde (NHSGGC), between April 2008 and April 2018, were identified from a prospectively maintained database. Those patients with a pre-operative ECG, pre-operative assessment of the systemic inflammatory response and had TNM stage I-III disease were assessed for inclusion. Exclusion criteria were as follows; patients without a pre-operative ECG, had no pre-operative assessment of the systemic inflammatory response or had TNM stage IV disease.

### Clinicopathological characteristics

Routine demographic details collected included age, sex and BMI. Age categories were grouped into < 64, 65–74 and > 74 years. Patient comorbidity was classified using the American Society of Anesthesiologists (ASA) grading system^20^. The presence of ischaemic heart disease, congestive heart failure and diabetes mellitus specifically was also identified from patients’ medical records^10,11^. The patient’s pre-operative medication history was identified from anaesthetic assessments and medical records. The cumulative anti-cholinergic burden (AChB) score of mediations was retrospectively calculated and grouped as < 3/ ≥ 3^21,22^. Tumour pathological characteristics including stage, differentiation and the presence of venous invasion were identified from pathology reports. Tumours were staged using the fifth edition of the TNM classification, consistent with practice current in the United Kingdom during the study period^23^.

The primary endpoint was overall survival (OS). The date and cause of death were confirmed using hospital electronic case records. Date of last recorded follow-up or last review of electronic case records was 21st March 2023, which served as the censor date. This retrospective observational study was approved by the West of Scotland Research Ethics Committee. Informed consent was obtained from all patients prior to surgery. All aspects of this study were performed in accordance with the Declaration of Helsinki.

### Heart rate variability (HRV)

Heart rate variability was analysed using a 12-lead, 10-s (150 Hz) pre-operative ECG performed within the 3 months prior to the date of surgery. If multiple ECG’s were available for the same, the most recent ECG before date of surgery was used for analysis. The time interval in milliseconds (ms) between consecutive R-peaks in lead II was used to calculate time-domain HRV parameters, Standard Deviation of NN-intervals (SDNN) and Root Mean Square of Successive Differences (RMSSD), as previously described^19^. The R-R time interval was calculated by manually counting the number of small boxes between R waves and multiplying the sum by 40 ms. Measurements of time intervals were performed by two individuals (JM and SL). The median values of SDNN and RMSSD were then calculated and used to categorised into low/high groups.

Patients with pre-operative ECG’s were excluded for the following reasons: patients with cardiac arrhythmias (including atrial and ventricular extrasystole), pacemakers, patients taking beta-blockers, patients with bradycardia (heart rate < 50 bpm) or tachycardia (heart rate > 110 bpm) as previously described^19,24^.

### Systemic Inflammation

The pre-operative systemic inflammatory response was determined using the Systemic Inflammatory Grade (SIG)- a combination of the neutrophil/lymphocyte ratio (NLR) and the modified Glasgow Prognostic Score (mGPS)^25^. Patients were categorised as grade 0–4, as follows: SIG 0 was defined as mGPS 0 and NLR < 3; SIG 1 as mGPS 0 and NLR 3–5 or mGPS 1 and NLR < 3; SIG 2 as mGPS 0 and NLR > 5 or mGPS 2 and NLR < 3 or mGPS 1 and NLR 3–5; SIG 3 as mGPS 1 and NLR > 5 or mGPS 2 and NLR 3–5 and SIG 4 as mGPS 2 and NLR > 5 (See Supplementary Table 1).

Pre‐operative haematological and biochemical results were identified from medical records and prospectively recorded. Blood samples were either obtained at pre-operative assessment, within 30 days of surgery, for elective patients or on admission for patients undergoing emergency surgery. An autoanalyzer was used to measure serum CRP (mg/L) and albumin (g/L) concentrations (Architect; Abbot Diagnostics, Maidenhead, UK).

### Statistical analysis

SDNN, RMSSD, TNM stage, ASA, SIG and OS were presented as categorical variables. Categorical variables were analysed using Chi-square test for linear-by-linear association. For categorical variables, Fisher’s exact test was used when value of single cell of a two-by-two table was n ≤ 5.

The prognostic value of SDNN and RMSSD to OS were examined using univariate and multivariate Cox’s proportional-hazards model. SDNN and RMSSD were presented as both continuous and categorical (median) variables. To examine the relationships between OS and clinicopathological variables, and adjust for potential confounding factors, the authors included any variable showing an association below the significance threshold of p < 0.10 on univariate analysis in the backward conditional multivariate model. A two-sided *p* value < 0.05 was considered statistically significant. OS was defined as the time (months) from date of surgery to date of death due to any cause.

Missing data were excluded from analysis on a variable-by-variable basis. Two-tailed *p* values < 0.05 were considered statistically significant. Statistical analysis was performed using SPSS software version 27.0 (SPSS Inc., Chicago, IL, USA).

## Results

### Patient inclusion

**Figure 1:**
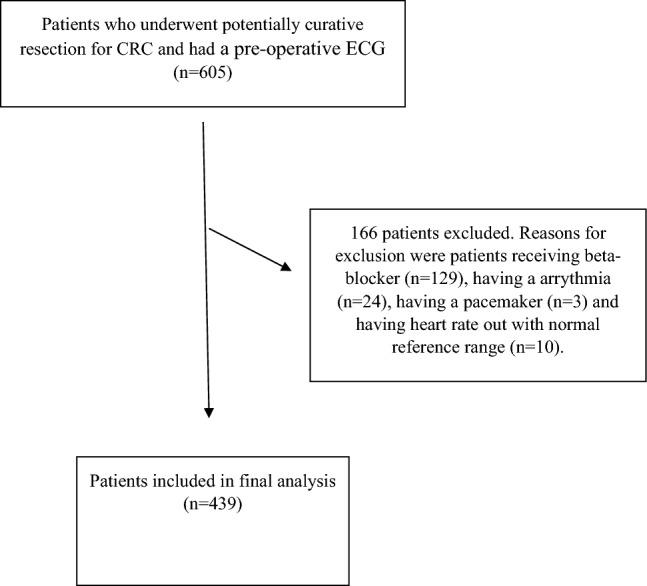
Flow diagram of included patients.

Of the 605 patients with CRC cancer, who underwent potentially curative resection during the study timeframe, 166 did not meet the inclusion criteria (see Fig. 1). The clinicopathological characteristics of the included patients are shown in Table 1. The median follow-up was 78 months (IQR 61–99 months) and 31% (n = 137) patients died during the follow-up (see Table 1).

**Table 1:** Clinicopathological characteristics of included patients (n = 439).

Clinicopathological Characteristic	n = 439 (%)
Age (years)	
< 65	189 (43%)
65–74	152 (35%)
> 74	98 (22%)
Sex	
Male	225 (51%)
Female	214 (49%)
Tumour site	
Colon	241 (55%)
Rectum	198 (45%)
TNM stage	
I	116 (26%)
II	158 (36%)
III	165 (38%)
Differentiation	
Moderate-Well	396 (90%)
Poor	43 (10%)
Venous invasion	
No	180 (41%)
Yes	259 (59%)
ASA	
1	117 (27%)
2	215 (49%)
≥ 3	107 (24%)
Ischaemic heart disease	
No	259 (59%)
Yes	180 (41%)
Heart failure	
No	433 (99%)
Yes	6 (1%)
Diabetes mellitus	
No	375 (85%)
Yes	64 (15%)
AChB score	
< 3	370 (84%)
≥ 3	69 (16%)
SIG	
0	192 (44%)
1	120 (27%)
≥ 2	127 (29%)
SDNN (ms)	
≥ 24.0	222 (51%)
< 24.0	217 (49%)
RMSSD (ms)	
≥ 29.8	226 (52%)
< 29.8	213 (48%)
OS	
Yes	302 (69%)
No	137 (31%)

Inter-rater reliability was assessed in a sample of 30 random patient ECG’s, with an inter-class correlation coefficient (ICC) of 1.000. The median SDNN was 24.0 ms (interquartile range, IQR, 17.9–35.8 ms) and 49% (n = 217) of patients were categorised as having a low SDNN (< 24.0 ms). The median RMSSD was 29.8 ms (IQR 23.1–42.2 ms) and 48% (n = 213) of patients were categorised as having a low RMSSD (< 29.8 ms).

The relationship between SDNN (median) and tumour pathology, co-morbidity, systemic inflammation and OS in patients undergoing potentially curative surgery for CRC is shown in Table 2. On univariate analysis, SDNN was significantly associated with RMSSD (p < 0.001). On univariate analysis, SDNN was not significantly associated with age (p = 0.180), sex (p = 0.539), tumour site (p = 0.867), TNM stage (p = 0.830), differentiation (p = 0.935), venous invasion (p = 0.052), ASA (p = 0.598), ischaemic heart disease (p = 0.557), heart failure (p = 0.445), diabetes mellitus (p = 0.921), AChB score (p = 0.071), SIG (p = 0.898) or OS (p = 0.882, see Table 2).

**Table 2:** The relationship between SDNN (median) and tumour pathology, co-morbidity, systemic inflammation and OS in patients undergoing potentially curative surgery for CRC (n = 439).

	SDNN ≥ 24.0 ms (n = 222)/ %	SDNN < 24.0 ms (n = 217)/ %	p value ^1^
Age (years)			0.18
< 65	107 (48%)	82 (38%)	
65–74	65 (29%)	87 (40%)	
> 74	50 (23%)	48 (22%)	
Sex			0.539
Male	117 (53%)	108 (50%)	
Female	105 (47%)	109 (50%)	
Tumour site			0.867
Colon	121 (55%)	120 (55%)	
Rectum	101 (45%)	97 (45%)	
TNM stage			0.83
I	58 (26%)	58 (27%)	
II	83 (37%)	75 (35%)	
III	81 (37%)	84 (39%)	
Differentiation			0.935
Moderate-well	200 (90%)	196 (90%)	
Poor	22 (10%)	21 (10%)	
Venous invasion			0.052
No	81 (37%)	99 (46%)	
Yes	141 (63%)	118 (54%)	
ASA			0.598
1	61 (28%)	56 (26%)	
2	109 (49%)	106 (49%)	
≥ 3	52 (23%)	55 (25%)	
Ischaemic heart disease			0.557
No	134 (60%)	125 (58%)	
Yes	88 (40%)	92 (42%)	
Heart failure			0.445
No	220 (99%)	213 (98%)	
Yes	2 (1%)	4 (2%)	
Diabetes mellitus			0.921
No	190 (86%)	185 (85%)	
Yes	32 (14%)	32 (15%)	
AChB score			0.071
< 3	194 (87%)	176 (81%)	
≥ 3	28 (13%)	41 (19%)	
SIG			0.898
0	94 (42%)	98 (45%)	
1	68 (31%)	52 (24%)	
≥ 2	60 (27%)	67 (31%)	
RMSSD (ms)			< 0.001
≥ 29.8	189 (85%)	37 (17%)	
< 29.8	33 (15%)	180 (83%)	
OS			0.882
Yes	152 (69%)	150 (69%)	
No	70 (31%)	67 (31%)	

^1^p value is from χ2 analysis or Fisher’s exact test when value of single cell n ≤ 5.

The relationship between RMSSD (median) and tumour pathology, co-morbidity, systemic inflammation and survival in patients undergoing potentially curative surgery for CRC is shown in Table 3. On univariate analysis, RMSSD was significantly associated with AChB score (p < 0.05) and SDNN (p < 0.001). On univariate analysis, RMSSD was not significantly associated with age (p = 0.700), sex (p = 0.323), tumour site (p = 0.183), TNM stage (p = 0.267), differentiation (p = 0.314), venous invasion (p = 0.061), ASA (p = 0.294), ischaemic heart disease (p = 0.219), heart failure (p = 1.000), diabetes mellitus (p = 0.172), SIG (p = 0.951) or OS (p = 0.319, see Table 3). When this analysis was repeated in AChB score < 3 patients were examined, there were no new significant results observed. Therefore, these results were not displayed in detail.

**Table 3:** The relationship between RMSSD (median) and tumour pathology, co-morbidity, systemic inflammation and survival in patients undergoing potentially curative surgery for CRC (n = 439).

	RMSSD ≥ 29.8 ms	RMSSD < 29.8 ms	p value ^1^
	(n = 226)/ %	(n = 213) / %	
Age (years)			0.7
< 65	104 (46%)	85 (40%)	
65–74	68 (30%)	84 (39%)	
> 74	54 (29%)	44 (21%)	
Sex			0.323
Male	121 (54%)	104 (49%)	
Female	105 (46%)	109 (51%)	
Tumour site			0.183
Colon	131 (58%)	110 (52%)	
Rectum	95 (42%)	103 (48%)	
TNM Stage			0.267
I	61 (27%)	55 (26%)	
II	88 (39%)	70 (33%)	
III	77 (34%)	88 (41%)	
Differentiation			0.314
Moderate-well	207 (92%)	189 (89%)	
Poor	19 (8%)	24 (11%)	
Venous invasion			0.061
No	83 (37%)	97 (46%)	
Yes	143 (63%)	116 (54%)	
ASA			0.294
1	64 (28%)	53 (25%)	
2	111 (49%)	104 (49%)	
≥ 3	51 (23%)	56 (26%)	
Ischaemic heart disease			0.219
No	127 (56%)	132 (62%)	
Yes	99 (44%)	81 (38%)	
Heart failure			1
No	223 (99%)	210 (99%)	
Yes	3 (1%)	3 (1%)	
Diabetes mellitus			0.172
No	192 (85%)	183 (86%)	
Yes	34 (15%)	30 (14%)	
AChB score			0.048
< 3	198 (88%)	172 (81%)	
≥ 3	28 (12%)	41 (19%)	
SIG			0.951
0	96 (43%)	96 (45%)	
1	68 (30%)	52 (24%)	
≥ 2	62 (27%)	65 (31%)	
SDNN (ms)			< 0.001
≥ 24.0	189 (84%)	33 (16%)	
< 24.0	37 (16%)	180 (84%)	
OS			0.319
Yes	156 (69%)	146 (69%)	
No	70 (31%)	67 (31%)	

^1^p value is from χ2 analysis or Fisher’s exact test when value of single cell n ≤ 5.

The relationship between OS and TNM stage, ASA, SIG, SDNN and RMSSD is shown in Table 4. On univariate analysis, age (p < 0.001), TNM stage (p < 0.001), ASA (p < 0.001) and SIG (< 0.001) were significantly associated with OS. On multivariate analysis, age (p < 0.001), TNM stage (p < 0.001), ASA (p < 0.05) and SIG (p < 0.05) remained significantly associated with OS (see Table 4). When this analysis was repeated in only patients with TNM stage III disease, this did not lead to any new significant results. Therefore, these results were also not displayed in detail.

**Table 4:** The relationship between OS and TNM stage, ASA, SIG, SDNN and RMSSD (n = 439).

	Univariate analysis		Multivariate analysis	
	Hazard ratio (95% Confidence Interval)	p-value	Hazard ratio (95% Confidence Interval)	p-value
Age (< 65/65–74/ > 74)	1.66 (1.34–2.05)	< 0.001	1.52 (1.22–1.89)	< 0.001
Sex (Female/male)	1.36 (0.97–1.91)	0.078	–	0.209
TNM stage (I/II/III)	1.77 (1.41–2.23|)	< 0.001	1.72 (1.36–2.17)	< 0.001
ASA (1/2/ ≥ 3)	1.65 (1.30–2.10)	< 0.001	1.37 (1.06–1.76)	0.015
SIG (0/1/ ≥ 2)	1.45 (1.19–1.77)	< 0.001	1.23 (1.00–1.50)	0.045
SDNN (continous)	1.00 (1.00–1.01)	0.329	–	–
Low SDNN (no/yes)	0.95 (0.68–1.32)	0.748	–	–
RMSSD (continous)	1.00 (1.00–1.01)	0.148	–	–
Low RMSSD (no/yes)	0.99 (0.71–1.39)	0.971	–	–

## Discussion

To our knowledge, the present study is the largest to date to examine HRV in patients undergoing potentially curative resections for CRC. Recognised prognostic factors including advanced age, TNM stage, ASA and SIG were all found to be determinants of survival in the present, well-defined cohort confirming the internal validity of observations. However, neither measure of HRV (SDNN or RMSSD) was significantly associated with advanced age, disease stage, co-morbidity, inflammatory status or survival. As such the results are not only informative to the utility of HRV as a prognostic host-assessment in patients potentially curative CRC, but to the basis of the relationship between HRV and survival outcomes in patients with CRC.

Despite a reported association with reduced HRV and CRC outcomes^15,18^, examination of the prognostic value to survival outcomes specifically is limited. Particularly in patients with potentially curative disease, with the majority of studies including only those with locally advanced or metastatic disease in cohorts also including other tumour types^8,9^. Therefore, the present negative results add to the existing literature and are consistent with the recent report of Strous and co-workers that found neither measure of HRV was associated with cancer-specific or overall survival in a cohort of 428 patients with primary, non-metastatic colorectal cancer^19^. The patients included in the two studies were similarly matched in age, sex, co-morbidity, stage of disease and survival. Furthermore, with proportion of patients categorised as having low SDNN and RMSSD (49% and 48%, respectively, in the present study vs. 51% and 54%, respectively, in the previous study by Strous and Co-workers). However, heterogeneity did exist between the studies, with Strous and co-workers employing threshold values to categorise patients into low/high HRV based on previous studies. Furthermore, Strous and co-workers reporting a lower median RMSSD (17.5 ms). Nevertheless, the SDNN and RMSSD values reported in the present study are in keeping with those of other contemporary studies of HRV in patients with CRC^16,18^ and of other cancer subtypes/disease stages^12,18^. Therefore, taken together the present evidence suggests that HRV may have limited prognostic value with respect to survival in patients undergoing potentially curative surgery for CRC.

While an association between time-domain HRV measures (SDNN and RMSSD) and survival outcomes in patients with cancer has been reported in systematic reviews^8,9^, the basis of this relationship remains unclear. One hypothesis is that the vagal nerve is responsible for neuro-immune modulation, improving survival outcomes in cancer by dampening the systemic inflammatory response^2,3^. A recent study of 272 patients with advanced pancreatic cancer by De Couck and co-workers reported that in patients with longer survival, HRV was inversely associated with a systemic inflammation (CRP)^12^. However, Cherifi and co-workers, in a study of 202 patients with mainly locally advanced or metastatic ovarian cancer, reported that a low HRV was independently associated with survival when adjusted for inflammatory response (Neutrophil: lymphocyte ratio)^26^. Furthermore, to date, the majority of studies that reported a significant association between HRV and survival outcomes in cancer have been of patients with locally advanced or metastatic disease^12,26–28^. Given the association of reduced HRV and advanced disease stage^13,29^, tumour stage may be a major confounding factor to the relationship between HRV and survival^9^. Indeed, the median SDNN (24.0 ms) and RMSSD (29.8 ms) reported in the present study were higher than those reported by Cherifi and co-workers (Median SDNN 11.1 ms and median RMSSD 11.5 ms^26^. Therefore, further study across a range of disease stages is required to delineate the potential prognostic value of HRV to survival outcomes in patients with cancer.

There are a number of limitations to the present study. Firstly, the present study was retrospective in nature and therefore may be subject to sample bias. Secondly, the present study only included patients who underwent potentially curative surgery for CRC. Further large comparative studies of HRV across TNM stages, including advanced disease, will be informative to the true relationship between HRV and survival in patients with CRC. Lastly, the present study measured the time-interval between heartbeats manually, which may introduce observer error. This may confound the present observations and explain the absence of an association between time-domain HRV parameters and age, systemic inflammation, cardiac disease and survival in the present study. Nevertheless, measurements using the present methodology showed excellent correlation between observers and SDNN and RMSSD values consistent with other contemporary studies of HRV in patients with CRC^16,18^. Further studies utilizing automated measurement of time-domain parameters should readily confirm the present observations.

In conclusion, HRV was not significantly associated with TNM stage, co-morbidity, systemic inflammation or survival in this well-defined cohort of patients undergoing potentially curative resection for CRC.

## Supplementary Information

Supplementary Information.

## Data Availability

Raw data will be made available on request to the senior author (DCM).
