# Supplementary material for: The relationship between heart rate variability and TNM stage, co-morbidity, systemic inflammation and survival in patients with primary operable colorectal cancer
Source: Sci Rep. 2023 May 19;13:8157. doi: 10.1038/s41598-023-35396-x (PMC10198985; doi:10.1038/s41598-023-35396-x)
Supplement: Supplementary file 1 — Supplementary Information. [file 41598_2023_35396_MOESM1_ESM.docx]

**Supplementary Table 1.** Calculation of the Systemic Inflammatory Grade (SIG)

| **Systemic Inflammatory Grade (SIG)** |  |
| --- | --- |
| SIG 0 | mGPS 0 and NLR < 3 |
| SIG 1 | mGPS 0 and NLR 3–5 or mGPS 1 and NLR < 3 |
| SIG 2 | mGPS 0 and NLR > 5 or mGPS 2 and NLR < 3 or mGPS 1 and NLR 3–5 |
| SIG 3 | mGPS 1 and NLR > 5 or mGPS 2 and NLR 3–5 |
| SIG 4 | mGPS 2 and NLR > 5 |
